# Doppler estimates of pulmonary vascular resistance to phenotype pulmonary hypertension in heart failure

**DOI:** 10.1007/s10554-019-01591-z

**Published:** 2019-05-23

**Authors:** Ashwin Venkateshvaran, Jasmin Hamade, Barbro Kjellström, Lars H. Lund, Aristomenis Manouras

**Affiliations:** 1 Department of Medicine, Karolinska Institutet, Solna, Stockholm, Sweden; 2 Heart and Vascular Theme, Karolinska University Hospital, Stockholm, Sweden; 3 Department of Clinical Physiology, Karolinska Institutet, Huddinge, Stockholm, Sweden; 4 Cardiovascular Research Unit, Norrbacka S1:02, Karolinska University Hospital, 17176 Stockholm, Sweden

**Keywords:** Post-capillary pulmonary hypertension, Doppler echocardiography, Heart failure

## Abstract

An accurate distinction between isolated post-capillary pulmonary hypertension (Ipc-PH) and combined post- and pre-capillary pulmonary hypertension (Cpc-PH) is integral to therapy and prognosis in heart failure (HF). This study aimed to compare the ability of four previously validated Doppler estimates of pulmonary vascular resistance (PVR_Doppler_) to distinguish Ipc-PH from Cpc-PH in a well-defined HF population. Consecutive subjects referred for HF assessment underwent standard echocardiography immediately followed by right heart catheterization (RHC). Subjects with atrial fibrillation, acute coronary syndrome, significant valvular disease or poor image quality were excluded. PVR_Doppler_ estimates were correlated with invasive PVR and agreement was studied using Bland–Altman analysis. Receiver operating characteristics analyses were performed to determine the ability of PVR_Doppler_ methods to identify PVR > 3WU. 55 HF subjects (58 ± 16 years, 55% Ipc-PH) were analyzed. PVR_Doppler_ estimates demonstrated weak to modest associations with invasive PVR. The Doppler method proposed by Abbas et al. demonstrated relatively strong discriminatory ability to distinguish Ipc-PH from Cpc-PH (AUC = 0.79; 95% CI 0.63–0.96; p = 0.001). However, Bland–Altman analysis revealed wide limits of agreement (bias = 0; SD = 1.83WU) and greater variability at higher mean PVR. Conclusions: PVR_Doppler_ estimates demonstrate reasonable ability to distinguish Ipc-PH from Cpc-PH but may not be reliable independent PH distinguishers in HF.

## Introduction

Subjects with heart failure (HF) commonly present with post-capillary pulmonary hypertension (PH), which is associated with poor prognosis [1, 2]. Based on severity of pulmonary vascular resistance (PVR), current guidelines classify the PH into: a) isolated post-capillary PH (Ipc-PH), characterized by pulmonary venous congestion with passive rise in pulmonary pressures and b) combined post- and pre-capillary PH (Cpc-PH) marked by additional superimposed pulmonary vascular functional and structural alterations [3]. An accurate distinction between these two PH subgroups is imperative to diagnosis, prognosis and optimal therapy as subjects with Cpc-PH demonstrate worse clinical outcome and higher mortality [4].

PVR measurement by right heart catheterization (PVR_RHC_) is the preferred method to distinguish PH phenotypes in HF. However, accurate noninvasive PVR estimates are highly desirable to minimize health costs, patient discomfort and exposure to radiation. In subjects with advanced HF, non-invasive assessment of PVR may also be useful in serial assessment of hemodynamic suitability for heart transplantation and for tailoring pharmacological therapy. Multiple methods to assess PVR by Doppler (PVR_Doppler_) have been proposed but are not widely utilized owing to inconclusive and conflicting results [5–10]. Further, no studies have specifically compared the discriminatory strength of PVR_Doppler_ methods to distinguish Ipc-PH from Cpc-PH in a well-defined HF population.

With the background, we aimed to explore the utility of Doppler methods to distinguish PH phenotypes in HF by comparing four validated PVR_Doppler_ methods with reference standard PVR_RHC_.

## Methods

### Patient population

All consecutive subjects referred to the Karolinska University Hospital for right heart catheterization (RHC) from February 2014 through February 2018 for the assessment of HF were screened for enrollment. All subjects were hemodynamically stable during assessment and medical therapy was suitably titrated. Subjects with acute coronary syndrome or cardiac surgery within a period of < 1 year prior to RHC, in atrial fibrillation or under pacemaker therapy, with significant concomitant valvular disease or poor image quality were excluded. The study was approved by the local ethics committee (DNR 2008/1695-31) and all patients provided written informed consent.

### Echocardiography

All patients underwent a standard echocardiogram in keeping with current guidelines [11] employing a Vivid E9 ultrasound system (GE Ultrasound, Horten. Norway) equipped with a 2.5 MHz matrix array transducer. 2D gray-scale images were acquired at 50–80 frames/s over three heart cycles. Doppler tracings were recorded using a sweep speed of 100 mm/s. All images were subsequently exported and analyzed offline (EchoPAC PC, version 11.0.0.0 GE Ultrasound, Waukesha, Wisconsin) by experienced operators blinded to catheterization data. Left ventricular ejection fraction (LVEF) was estimated using the Simpson’s biplane method. PVR_Doppler_ were calculated employing four different methods as previously described [5–8]. To estimate PVR_Doppler_ in the two methods proposed by Abbas et al. [5, 6], peak tricuspid regurgitation velocity (TRV_max_) was measured considering the most optimal signal obtained using apical and parasternal windows and right ventricular outflow tract velocity time index (RVOT_VTI_) was obtained by placing a 5 mm pulse-wave (PW) Doppler sample volume proximal to the pulmonary valve in the right ventricular outflow tract (RVOT) and tracing the resultant spectral wave form. For the method proposed by Scapellato et al. [7], pre-ejection period (PEP) was defined as time between QRS-start and RVOT-onset. Acceleration time (AcT) was expressed as the time between RVOT-onset and the RVOT-peak while total time (TT) was expressed as time interval between RVOT-onset and the RVOT-end. Finally, to estimate PVR using the method proposed by Haddad et al. RVOT_VTI_ was measured as previously described, systolic pulmonary artery pressure (SPAP) was obtained by adding the recommended estimates of RA pressure [11] to the corresponding pressure gradient obtained from the TRV_max_ signal.

### Right heart catheterization

RHC was performed using a 6F Swan Ganz catheter employing jugular or femoral vein access within 1 h post echocardiographic evaluation. Mean right atrial pressure (RAP_M_), systolic-, diastolic- and mean pulmonary artery pressure (PAP_S_, PAP_D_, PAP_M_) and mean pulmonary arterial wedge pressure (PAWP_M_) were obtained under fluoroscopic guidance after calibration with the zero-level set at the mid-thoracic line. Oxygen consumption was measured breath-by-breath by dedicated gas analysis system. Cardiac output (CO) was measured using the Fick’s principle. PVR_RHC_ was calculated as PVR_RHC_ = (PAP_M _− PAWP_M_)/CO, transpulmonary gradient (TPG) as TPG = PAP_M _− PAWP_M_ and diastolic pulmonary gradient (DPG) as DPG = PAP_D _− PAWP_M_. Post-capillary PH was defined as RHC derived PAP_M_ ≥ 25 mmHg and PAWP_M_ > 15 mmHg. Patients were further divided into Ipc-PH (PVR ≤ 3 and/or DPG < 7) and Cpc-PH (PVR > 3 and/or DPG ≥ 7) in keeping with current recommendations [3]. All pressure tracings were stored (WITT Series III, Witt Biomedical Corp., Melbourne, FL) and analyzed off-line by one experienced operator.

### Statistical methods

Normality was tested using the Shapiro–Wilk test and visually reaffirmed using QQ plots. Continuous variables are expressed as mean ± SD or median and interquartile range. Categorical variables are expressed as numbers and percentage. Ipc-PH and Cpc-PH subgroups were compared using two-sample Student’s or Man–Whitney test. Correlations between echocardiographic and invasive measurements were performed using the Pearson’s 2-tailed test. Multivariate regression analysis was performed to study association of PVR_Doppler_ estimates with PVR_RHC_ when adjusted for confounders. Receiver operator characteristics (ROC) analysis was performed to determine the discriminatory ability of the selected Doppler method to identify elevated PVR (> 3WU). Statistical significance of the difference between the areas of multiple ROC curves was calculated using the method of Delong et al. [12]. Regression equations were derived for each of the Doppler-derived PVR methods and agreement between echocardiographic and invasive PVR was showcased using Bland–Altman plots. Tests were performed at 95% confidence intervals and a p value ≤ 0.05 was considered statistically significant. To evaluate the intra- and inter-observer variability of the methods, ten subjects were measured twice by the same investigator and by two different investigators and interclass correlation coefficient for absolute agreement and mean value and standard deviation of differences were calculated. All methods demonstrated adequate to good feasibility and excellent intra-observer and inter-observer concordances (Table 1). IBM SPSS statistics version 23.0 was employed for analysis.

**Table 1:** Feasibility and reproducibility of Doppler-based PVR methods

PVR_Doppler_ methods	Feasibility (%)	Inter-class correlation	CI	Intra-class correlation	CI
Abbas et al. [5]	86	0.98	0.95–0.99	0.99	0.97–0.99
Abbas et al. [6]	86	0.98	0.96–0.99	0.99	0.98–0.99
Scapellato et al. [7]	93	0.93	0.90–0.95	0.95	0.93–0.97
Haddad et al. [8]	86	0.98	0.94–0.99	0.99	0.97–0.99

## Results

**Fig. 1:**
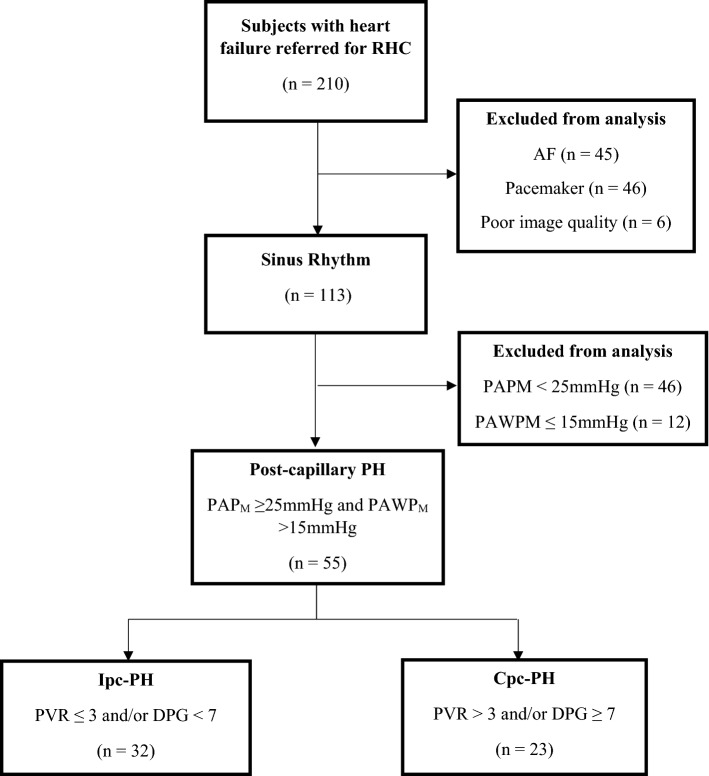
Patient flow chart

Of 210 subjects referred for RHC during the study period, 55 fulfilled criteria and were included in the analysis (Fig. 1). Patient characteristics are presented in Table 2. All subjects demonstrated signs and symptoms of HF, elevated NTproBNP and objective evidence of ventricular systolic and/or diastolic dysfunction. Thirty-two subjects (58%) were classified as Ipc-PH and 23 (42%) as Cpc-PH (Table 2). More than half the subjects demonstrated a LVEF < 50% (n = 30; 54%).

**Table 2:** Characteristics of patient population

	All (n = 55)	Ipc-PH (n = 32)	Cpc-PH (n = 23)	p-Value
Demographics				
Age (years)	58 ± 16	54 ± 15	63 ± 17	**0.03**
Female	18 (33)	5 (15)	13 (56)	**< 0.001**
Medical history				
Diabetes	15 (27)	10 (31)	5 (21)	0.48
Hypertension	30 (54)	17 (53)	13 (56)	0.68
Hypercholesteremia	17 (30)	7 (21)	10 (43)	0.18
NYHA class				
I/II/III/IV	2/7/42/4 (4/13/76/7)	2/6/21/3 (6/18/65/9)	0/1/21/1 (0/4/92/4)	0.17
Clinical assessment				
HR (bpm)	67 (60;82)	66 (60;79)	70 (60;88)	0.43
BSA (m^2^)	1.9 ± 0.2	2.0 ± 0.2	1.9 ± 0.2	**0.02**
SBP (mmHg)	112 ± 26	111 ± 26	114 ± 26	0.67
DBP (mmHg)	64 ± 14	62 ± 12	68 ± 17	0.19
Laboratory				
NTproBNP (ng/L)	2480 (1310;3110)	2480 (1310;2930)	2385 (855;3507)	0.95
Hb (g/L)	129 ± 21	132 ± 22	124 ± 18	0.16
Serum creatinine (μmol/L)	105 (85;130)	106 (85;130)	105 (84;130)	0.62
Diagnosis				
RCM	6 (11)	4 (13)	2 (9)	
DCM	6 (11)	5 (16)	1 (4)	
HCM	4 (7)	3 (9)	1 (4)	
ICM	20 (36)	9 (28)	11(48)	
Multifactorial	18 (33)	10 (31)	8 (35)	
Myocarditis	1 (2)	1 (3)	0 (0)	

Bold text was chosen to emphasize that these values were significant (*p* < 0.05)

Data presented as mean ± SD/median (Q1;Q3) or number (%). P-value represents difference between Ipc-PH and Cpc-PH

*Ipc-PH* isolated post-capillary pulmonary hypertension (PH), *Cpc-PH* combined post- and pre-capillary, *NYHA* New York heart association function class, *HR* heart rate, *BSA* body surface area, *SBP* systolic blood pressure, *DBP* diastolic blood pressure, *NTproBNP* N-terminal pro b-type natriuretic peptide, *Hb* hemoglobin, *RCM* restrictive cardiomyopathy, *DCM* dilated cardiomyopathy, *HCM* hypertrophic cardiomyopathy, *ICM* ischemic cardiomyopathy

Patients with Cpc-PH were older, more often female (Table 2) and had higher PA pressures, PVR and lower CO (Table 3) than patients with Ipc-PH. Echocardiographic evaluation revealed no differences in LVEF or ventricular dimensions between the two groups. PVR echo surrogates proposed by Abbas et al. [5, 6]. and Haddad et al. [8]. were higher in Cpc-PH than in Ipc-PH while the method proposed by Scapellato et al. [7]. showed no difference between groups (Table 3). Eight subjects (14%) demonstrated no or insignificant TR, 27 subjects (50%) demonstrated mild TR, 17 (31%) moderate and 3 (5%) had severe TR.

**Table 3:** Invasive and echocardiographic data of patient population

	All post-capillary PH (n = 55)	Ipc-PH (n = 32; PVR ≤ 3 and/or DPG < 7)	Cpc-PH (n = 23; PVR > 3 and/or DPG ≥ 7)	p-Value
Right heart catheterization				
PAWP_M_ (mmHg)	24 ± 6	24 ± 6	25 ± 6	0.98
PAP_S_ (mmHg)	58 ± 15	52 ± 12	66 ± 15	**< 0.001**
PAP_D_ (mmHg)	24 ± 7	23 ± 6	26 ± 8	**0.04**
PAP_M_ (mmHg)	38 ± 9	34 ± 7	43 ± 10	**0.001**
RAP (mmHg)	12 ± 6	12 ± 6	12 ± 6	0.98
PVR (WU)	3.3 ± 2.1	2.0 ± 0.7	5.2 ± 2.0	**< 0.001**
TPG (mmHg)	13 ± 6	10 ± 3	18 ± 6	**< 0.001**
CO (L/min)	4.4 ± 1.5	5.1 ± 1.6	3.6 ± 0.9	**< 0.001**
Echocardiography				
LVIDd (mm)	57 ± 13	58 ± 11	54 ± 14	0.28
LVEF (%)	44 ± 22	40 ± 19	46 ± 18	0.27
LVEF < 50% (n)	30 (54)	19 (34)	11 (20)	0.32
RVIDd (mm)	43 ± 8	43 ± 8	43 ± 7	0.88
RA Area (cm^2^)	22 ± 8	16 ± 6	22 ± 9	0.65
TAPSE (mm)	16 ± 6	16 ± 6	16 ± 6	0.73
TRV_max_ (m/s)	3.1 ± 0.7	3.0 ± 0.6	3.3 ± 0.9	**0.002**
RVSP (mmHg)	56 ± 17	49 ± 12	64 ± 21	**0.002**
E/e’septal	17 ± 9	17 ± 9	18 ± 9	0.74
LAVI(ml/m^2^)	54 ± 14	54 ± 15	55 ± 16	0.86
PVR_Doppler_ methods				
Abbas et al. [5]	0.22 ± 0.11 999.00	0.18 ± 0.08	0.30 ± 0.13	**< 0.001**
Abbas et al. [6]	0.75 ± 0.48	0.56 ± 0.28	1.00 ± 0.6	**< 0.001**
Scapellato et al. [7]	0.003 ± 0.001	0.003 ± 0.001	0.004 ± 0.002	0.13
Haddad et al. [8]	0.05 ± 0.03	0.04 ± 0.02	0.08 ± 0.04	**0.01**

Bold text was chosen to emphasize that these values were significant (*p* < 0.05)

Data presented as mean ± SD or number (%). P-value represents difference between Ipc-PH and Cpc-PH

*PH* pulmonary hypertension, *Ipc-PH* isolated post-capillary pulmonary hypertension, *Cpc-PH* combined post- and pre-capillary pulmonary hypertension, *PAWP*_*M*_ mean pulmonary capillary wedge pressure, *PAP* pulmonary artery pressure, *s* systolic, *d* diastolic, m mean, *RAP* right atrial pressure, *PVR* pulmonary vascular resistance, *TPG* transpulmonary gradient, *CO* cardiac output, *LVIDd* left ventricular internal diameter during end-diastole, *EF* ejection fraction, *RVID* right ventricular internal diameter end-diastole, *RA* right atrium, *TAPSE* tricuspid annular plane systolic excursion, *TRV*_*max*_ tricuspid regurgitation max velocity, *RVSP* right ventricular systolic pressure, *E/e′* transmitral early diastolic to myocardial early diastolic velocity ratio, *LAVI* left atrial volume index, *RVOT*_*VTI*_ velocity time integral across right ventricular outflow tract, *PEP* pre-ejection period, *AcT* acceleration time, *TT* total time, *SPAP* systolic pulmonary artery pressure

### Association between echocardiographic and invasive PVR estimates

PVR_Doppler_ methods proposed Abbas et al. [5, 6] and Haddad et al. [8]. demonstrated significant associations with invasive PVR. Of the four methods, The revised method proposed by Abbas et al. [6]. demonstrated the strongest association with invasive PVR (Table 4) and was an independent predictor of invasive PVR as per the regression equation PVR_Abbas_ = 1.31 + 2.59x [(TRVmax)^2^/VTI]. Even when adjusted for age, gender and BMI, this method continued to demonstrate strongest association with PVR_RHC_ (β = 0.63, p < 0.001). No significant correlations were observed between Doppler-derived and invasive PVR when Cpc-PH patients were separately analyzed (p > 0.05). Comparison between echocardiographic and corresponding RHC variables demonstrated a weak association between RVOT_VTI_ and Fick-derived CO (r = 0.38; p < 0.01) and modest relationship between TRV_max_ and invasive PAP_s_ (r = 0.52; p < 0.001).

**Table 4:** Correlations between invasive and Doppler-based PVR

PVR_Doppler_ methods	PVR formula	R value	P value
Abbas et al. [5]	TRV_max_/RVOT_VTI_	0.52	<0.001
Abbas et al. [6]	TRV_max_^2^/RVOT_VTI_	0.57	<0.001
Scapellato et al. [7]	(PEP/AcT)/TT	0.37	0.06
Haddad et al. [8]	SPAP/(HR × RVOT_VTI_)	0.50	<0.001

### PVR_Doppler_ ability to distinguish PH phenotypes

**Fig. 2:**
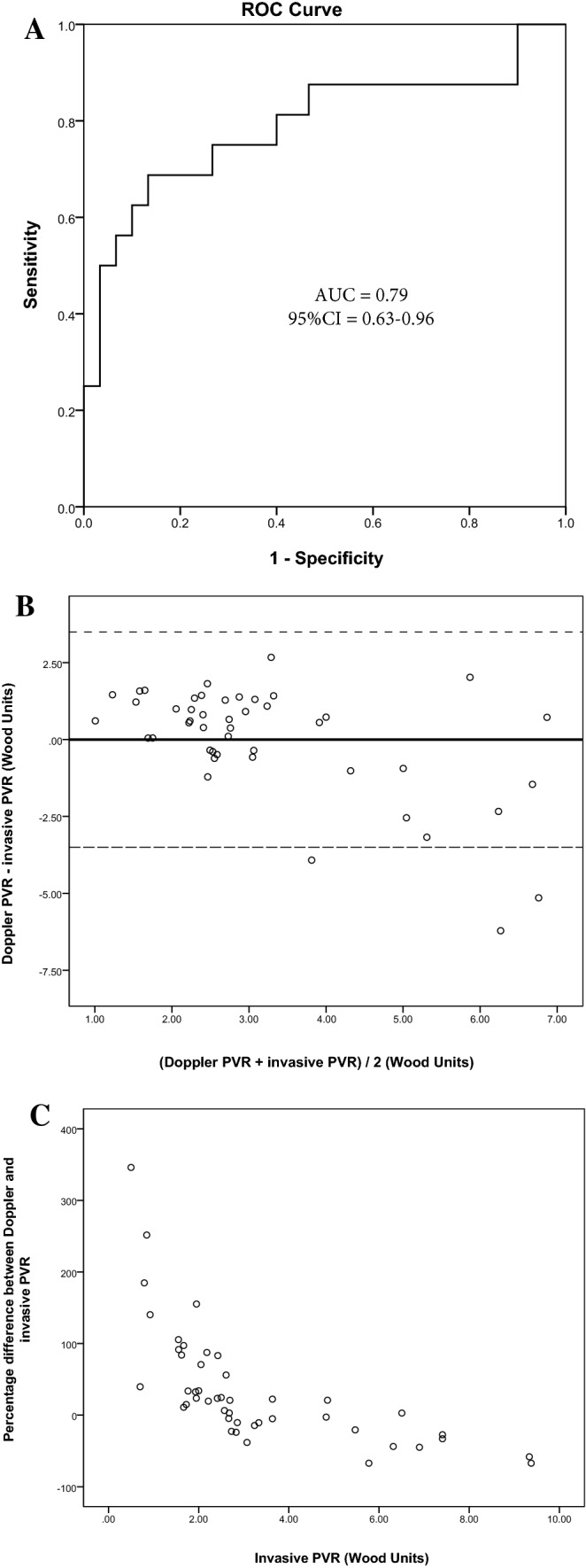
Diagnostic accuracy of Doppler-derived PVR as per method proposed by Abbas et al. **a** Receiver-operating characteristic curve. A TRV_max_^2^/TVI_RVOT_ cut-off value of 0.59 provided best balanced sensitivity (81%) and specificity (65%) to determine PVR > 3WU. **b** Bland–Altman analysis of PVR obtained by Doppler and invasive PVR. **c** Percentage difference between Doppler-derived and invasive PVR plotted against invasive PVR

PVR_Doppler_ by methods proposed by Abbas et al. [5, 6] and Haddad et al. [8] demonstrated a fair discriminatory ability to distinguish Ipc-PH from Cpc-PH subjects in our patient group (Table 5). Of these three, the revised method proposed by Abbas et al. [6] demonstrated highest area under the curve (AUC = 0.79; 95% CI 0.63–0.96; p = 0.001) (Table 5 and Fig. 2a). A cut-off value of 0.59 provided best balanced sensitivity (81%) and specificity (65%) to determine PVR > 3WU. Bland–Altman analysis, however, revealed wide limits of agreement between RHC and Doppler-based PVR (bias = 0; SD = 1.83WU) and greater variability was observed with higher PVR (Fig. 2b). Further, when the difference between PVR_Doppler_ and PVR_RHC_ was expressed as percentage and plotted against PVR_RHC_, a large relative difference was observed in the setting of non-elevated PVR (Fig. 2c). In patients with PVR ≤ 3WU, Bland–Altman demonstrated better limits of agreement but greater bias with Doppler-based PVR demonstrating higher values as compared with invasive measurements (Bias = 0.8; SD = 0.76WU). No significant difference was observed when comparing AUC curves between PVR_Doppler_ methods proposed by Abbas et al. [5, 6] and Haddad et al. [8] (p > 0.05 for all comparisons).

**Table 5:** Discriminatory ability of Doppler-based PVR methods to distinguish Ipc-PH and Cpc-PH and Bland–Altman analysis (n = 55)

PVR_Doppler_ methods	PVR formula	AUC	CI	P value	Bland–Altman mean difference ± SD
Abbas et al. [5]	TRV_max_/RVOT_VTI_	0.77	0.60–0.93	0.003	0.02 ± 1.89
Abbas et al. [6]	TRV_max_^2^/RVOT_VTI_	0.79	0.63–0.96	0.001	0.00 ± 1.83
Scapellato et al. [7]	(PEP/AcT)/TT	0.60	0.44–0.76	0.195	0.00 ± 2.01
Haddad et al. [8]	SPAP/(HR × RVOT_VTI_)	0.72	0.54–0.86	0.012	0.00 ± 1.91

## Discussion

The ability of Doppler derived methods to assess PVR has been debated. Early studies focused on pulmonary artery morphological flow patterns demonstrated poor association with invasive data [13, 14]. More recently, investigations incorporating Doppler surrogates of TPG and CO have exhibited greater potential to accurately represent PVR_RHC_ in pulmonary arterial hypertension [8, 10] cardiomyopathy [15, 16], chronic HF with severe systolic dysfunction [7] and mixed PH populations [5, 6]. To our knowledge, a comparison between multiple Doppler assessments to specifically distinguish Ipc-PH from Cpc-PH in the context of HF has not been performed.

Abbas et al. in their first study suggested that the relationship between TRV_max_ and VTI_RVOT_—noninvasive surrogates of right sided pressure and flow demonstrated a strong correlation and satisfactory limits of agreement with invasively obtained PVR [5]. As compared with that study, our patient group demonstrated more severe PVR, higher PA pressures and lower EF suggesting a more severe clinical presentation. Scapellato and colleagues enrolled subjects with chronic HF in their validation effort [7]. While all their subjects presented with severely impaired LV systolic performance, a closer inspection of the hemodynamic profile of that group reveals less severe degrees of pulmonary vascular derangements as compared with our cohort. The generally weaker associations with invasive measurements exhibited by the aforementioned methods in our cohort may partially be attributed to less severe clinical presentations, and to the inherent limitations of these methods to assess higher PVR [9].

To address these limitations, Haddad et al. [8], and in a revised version, Abbas et al. [6] chose to validate newer Doppler methods aimed at subjects with more severe PVR elevation. While these two methods demonstrated relatively stronger correlation with invasive PVR, the strength of association was modest. Both Haddad et al. [8]. and Abbas et al. [6]. incorporated TVI_RVOT_ and TRV_max_ in their equations, which demonstrated only modest correlations with corresponding invasive measurements. While the reason for a lack of strong association is unclear, one can speculate that the determination of TRV_max_ may be a potential source of error in the specific setting of high PVR. In the simplified Bernoulli’s equation, pulmonary artery systolic pressure gradient is estimated by squaring TRV_max_. Given this relationship, small changes in TRV_max_ will likely reflect larger changes in PA pressures in subjects with more severe PH as compared with those with no or mild PH, predisposing this method to greater error. The lack of association between Doppler-derived and invasive PVR in the specific context of Cpc-PH in our cohort further strengthens this speculation. Further, the absence of measurable TR does not necessarily rule out significant PH [17], suggesting that overt dependency on this echocardiographic component to determine possible elevations in PVR may not always be reliable.

The identification of Doppler methods that accurately distinguish Ipc-PH from Cpc-PH is particularly attractive in HF, as a surrogate for RHC during screening for HF device therapies and heart transplantation, assessments of pharmacological therapy and to predict outcomes. In the present study, Doppler methods demonstrated reasonable ability to distinguish these two PH groups but may be not be reliable independent diagnostics. Even the best performing of the four tested Doppler indices [6] demonstrated no significant associations in the setting of elevated PVR, had wide limits of agreement with invasive measurements and greater variability as mean PVR increased. These results suggest that the use of Doppler indices in the absence of RHC, as has been proposed in previous studies [18, 19] might not be advisable. Continued search for an accurate noninvasive measure in the specific context of HF with significant PH is warranted.

Strengths of the study include data acquisition where catheterization was performed within 1 h after echocardiography, minimizing the probability of significantly altered hemodynamic states. Limitations include the assessment of CO using only the Fick method, while a comparison with the measurement of CO by thermodilution may have added value. Additionally, the exclusion of subjects owing to insignificant TR signals could have been avoided with administration of agitated saline contrast.

## Conclusion

While Doppler-based assessments of PVR demonstrate reasonable ability to distinguish Ipc-PH from Cpc-PH, wide limits of agreement with invasive measurements and greater variability at higher PVR preclude the wider utilization of these methods as reliable independent PH distinguisher in the setting of HF.
